# Inconsistent Findings of Cardiac Troponin T and I in Clinical Routine Diagnostics: Factors of Influence

**DOI:** 10.3390/jcm10143148

**Published:** 2021-07-16

**Authors:** Abass Eidizadeh, Laura Fraune, Andreas Leha, Rolf Wachter, Abdul R. Asif, Lutz Binder

**Affiliations:** 1 Institute for Clinical Chemistry/Interdisciplinary UMG Laboratory, University Medical Center Goettingen, 37075 Goettingen, Germany; laura.fraune@yahoo.de (L.F.); asif@med.uni-goettingen.de (A.R.A.); lutz.binder@med.uni-goettingen.de (L.B.); 2 Department of Medical Statistics, University Medical Center Goettingen, 37073 Goettingen, Germany; andreas.leha@med.uni-goettingen.de; 3 German Centre for Cardiovascular Research (DZHK), 37075 Göttingen, Germany; wachter@med.uni-goettingen.de; 4 Clinic and Policlinic for Cardiology, University Hospital Leipzig, 04103 Leipzig, Germany; 5 Clinic for Cardiology and Pneumology, University Medical Center Goettingen, 37075 Goettingen, Germany

**Keywords:** troponin I, troponin T, comparison, discrepancy, gender, cutoffs, 99th percentile upper reference limit, sex-specific, myocardial infarction, cardiac biomarker

## Abstract

Cardiac troponins are crucial for the diagnosis of acute myocardial infarction. Despite known differences in their diagnostic implication, there are no recommendations for only one of the two troponins, cardiac troponin I (cTnI) and troponin T (cTnT) so far. In an everyday routine diagnostic, cTnT (Roche) as well as cTnI (Abbott) were measured in 5667 samples from 3264 patient cases. We investigated the number of identical or discrepant troponin findings. Regarding cTnI, we considered both, sex-dependent and unisex cutoffs. In particular, the number of cTnT positive and cTnI negative results was strikingly high in 14.0% of cTnT positive samples and increases to 23.8% by using sex-specific cTnI cutoffs. This group was considerably greater than the group of cTnI positive and cTnT negative results, also after elimination of patients with an eGFR < 60 mL/min/1.73 m^2^. Comparing the troponin cases with a dynamic increase or decrease between two measurements, we saw a balanced number of discrepant cases (between cTnT+/cTnI− and cTnT−/cTnI+), which was, however, still present. Using ROC analysis, sex-dependent cutoffs improved sensitivity and specificity of cTnI. This study shows in a large cohort that comparing the two cardiac troponins does not amount to identical analytical results. Consideration of sex-dependent cutoffs may improve sensitivity and specificity.

## 1. Introduction

Acute myocardial infarction (AMI) is a life-threatening disorder that requires quick and safe diagnosis and therapy. Cardiac biomarkers are crucial for diagnosis, progress monitoring and prognosis [1]. The fourth universal definition of myocardial infarction names cardiac troponins (cTn) as decisive laboratory parameters in AMI diagnostics [2].

The troponin complex consists of three subunits (troponin C, troponin I, troponin T) and controls the muscular force by regulating the contractile apparatus in skeletal and cardiac muscle [3]. Troponin I (TnI) and troponin T (TnT) proteins occur as cardiac muscle isoforms. Commercial assays for measurement of cardiac isoforms in blood plasma have been available since the 1990s [4]. Plasma concentrations correlate with ischemic or non-ischemic myocardial injury and show high tissue specificity. Increases in troponin concentrations can be detected at 2 to 4 h after an ischemic event [5]. The high level of specificity made them fundamental for clinical diagnostics. At least one cTn value above the 99th percentile upper reference limit (URL) of a healthy reference population is considered as a criterion for myocardial injury. A significant decrease or increase of troponin plasma concentrations in serial measurements defines acute myocardial damage, and the parallel presence of clinical symptoms or ECG (electrocardiogram) changes defines the acute myocardial infarction [2]. The definitions refer to high-sensitive cardiac troponin assays (hs-cTn) which are recommended for routine use [6]. To date, however, there is no preference for one of the two relevant troponin proteins, although differences in biochemistry and differential diagnosis between elevated cardiac troponin I and troponin T, respectively, are well known [7,8,9,10,11,12]. Moreover, the altered kinetics of cardiac troponins in renal insufficiency may give rise to clinical misinterpretation [13]. Furthermore, there is an increasing number of studies suggesting the use of gender-specific cutoffs for cTn [14]. There are differences in the 99th percentile URL between men and women in healthy reference populations. So far, manufacturers have given gender-specific URLs for cTnI [15] only, but not for cTnT.

In this retrospective study on prospectively collected data, we wanted to work out differences between cTnI and cTnT results and with respect to the final diagnosis AMI. For this purpose, concentrations of both cTnT (Roche) and cTnI (Abbott) were measured in all patient blood samples with cTn concentration request. Furthermore, we determined at least one creatinine value for each patient case. Results of the 5667 blood samples from 3264 patient cases were analyzed for the following aspects: (I) How many discrepant positive or negative cTnT and cTnI results are found when using alternatively gender-independent or gender-specific upper limits of the cTnI reference range? How are the rate of the discordances between cTnT and cTnI changes through elimination of patients with an eGFR < 60 mL/min/1.73 m^2^? (II) How many discrepant findings exist when comparing serial troponin measurements under consideration of marker kinetics and clinical diagnosis? (III) How does gender-specific cutoffs and kidney function influence specificity and sensitivity of the two cTn?

## 2. Materials and Methods

### 2.1. Study Design

**Figure 1:**
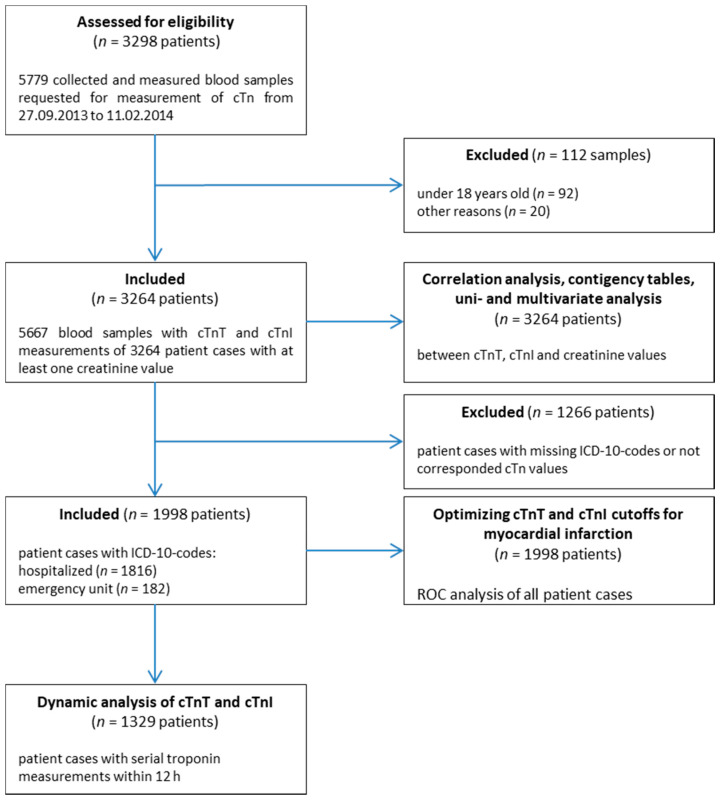
Study flow chart. This flow chart shows the sequence of the selection of the subgroups for the statistical analyzes. Of the 5785 originally measured samples, 5667 were included in the statistical analysis. Of these, subgroups were determined for the ROC (receiver operating characteristic) analyses (*n* = 1998) as well as for the dynamic analyses (*n* = 1329).

The differences between cTnT and cTnI should be worked out in a direct retrospective comparison. For this purpose, both cTnT and cTnI were measured promptly in all blood samples for which a troponin measurement was requested from the attending physician in the period from 27 September 2013 to 11 February 2014 in the central laboratory of the University Medical Center Göttingen (UMG). A total of 5779 samples were processed in this way (Figure 1). A total of 112 samples were excluded from the study due to technical failure (missing data transmission, no correct measurements, too little sample volume, etc.) or patients being under the age of 18 years (*n* = 92). The remaining 5667 sample results were included in the study and statistically analyzed. At least one creatinine value was recorded for each patient case (*n* = 3264) at the same time, when cTn was measured. The statistical analyses were either sample-related or patient-specific. For the ROC (receiver operating characteristic) analyzes, the ICD-10 (international statistical classification of diseases and related health problems) codes of the main diagnoses of the patient cases, available in the hospital accounting, were used. A total of 1266 patient cases could not be used due to the lack of any diagnostic data in our system. However, 1998 patient cases (1816 hospitalized, 182 from the emergency unit) could be used for the ROC analyzes. There were 1329 patient cases with an available diagnosis and at least two troponin measurements within 12 h that could be used for the dynamic analysis. The venous blood was analyzed from lithium-heparinate plasma (S-Monovette Li-Heparin, Sarstedt AG & Co., Nürnbrecht, Germany). The samples were centrifuged for 10 min at 4230 rpm at 20 °C. cTnT and cTnI were measured in succession. The study was conducted according to the World Medical Association Declaration of Helsinki and approved by the UMG Ethics Committee.

### 2.2. Cardiac Troponin Measurements

High-sensitive cardiac troponin T (cTnT) was measured from lithium-heparinate plasma on the Roche Cobas e411 using the Cobas Troponin T hs assay (Roche Diagnostics GmbH, Mannheim, Germany), an electrochemiluminescent immunoassay. The limit of detection was set at 5 ng/L according to the manufacturer’s instruction. The cutoff of this assay is given by the manufacturer as 14 ng/L. Values >14 ng/L were declared as positive.

High-sensitive cardiac Troponin I (cTnI) was measured from lithium-heparinate plasma using the Architect STAT High Sensitive Troponin-I Assay (Abbott, Langford, Ireland) on the Abbott Architect i2000SR. The assay is a chemiluminescent-microparticle immunoassay with a limit of detection of 1.9 ng/L. The manufacturer specifies a gender-independent cutoff (26.2 ng/L) and gender-dependent cutoffs (women: 15.6 ng/L; men: 34.2 ng/L). In this study, the effects of choosing a gender-specific vs. gender-independent cutoff should be examined. Therefore, all statistical analyses were carried out with both the gender-specific and unisex cutoffs of the manufacturer.

### 2.3. Creatinine and eGFR Measurements

Only patient cases with at least one measured creatinine value were included in the statistical analysis. Creatinine was measured in lithium-heparinate plasma using the Creatinine PAP FS assay from DiaSys (Holzheim, Germany) on the Architect c-16000 (Abbott, Chicago, IL, USA). The limit of detection was set at 0.1 mg/dL according to the manufacturer’s instruction. The estimated glomerular filtration rate (eGFR) was determined by the CKD-EPI (chronic kidney disease epidemiology collaboration) formula using the patient’s age and gender [16]. Based on the eGFR, six grades of impaired renal function were assigned [16].

### 2.4. Statistical Analysis

In addition to the measured parameters, age, gender and time of analysis were recorded for all patient cases. The descriptive data are given as absolute and relative frequencies for measured values, otherwise as median with minimum and maximum. In order to investigate the influence of the individual factors on the cTn concentrations, univariate linear mixed effect models as well as a multiple linear mixed effect model were fit to the data.

Troponin measurements were log scaled and values below the level of detection were imputed using model-based ordinary and robust expectation-maximization algorithms for imputation of left-censored values via coordinates representation of compositional data which incorporate the information of the relative covariance structure [17].

Linear correlations between cTnT and cTnI, as well as the creatinine value, were assessed and quantified using Pearson’s correlation coefficient (r). Furthermore, the cTn results were determined as positive or negative according to the cutoff, which was used and summarized in contingency tables. This was done sample-related.

The kinetics of the cTn concentration in patient blood is decisive for the clinical assessment [2]. Dynamic analyses were therefore carried out. Only patient cases with at least two serial troponin-measurements within 12 h were included (*n* = 1329). A patient case was defined as positive according to cTnI or cTnT or both if there were at least two measurements within 12 h, one concentration was above the chosen threshold and one of the measurements showed a difference of at least 20% compared to the first measurement. The results were compiled in contingency tables. In addition, based on the existing diagnoses, it was assigned for the patient cases whether a myocardial infarction was present or not.

ROC (receiver operating characteristic) analyses were conducted to differentiate myocardial infarction cases from non-myocardial-infarction cases using cTnT or cTnI in several subgroups. The ICD-10 coded main diagnoses of the patient cases, which were obtained from hospital accounting, served as basis (*n* = 1816). The ICD codes with subcategories of I21, I22 and I24 were considered as myocardial injury. The thresholds corresponding to the Youden index were calculated. The discrimination ability was assessed via the AUC (area under the curve). ROC curves were compared using DeLong’s test. In order to find a discriminatory cutoff that discriminates early, the first cTn measurement above the established cutoff was used. If all measurements were below the established cutoff, the maximal cTn result was used.

The significance level was set to α = 5% for all statistical tests. All analyses were performed with the statistic software R (version 3.5.2; R Core Team 2018, Vienna, Austria) using the R-package lme4 [18] (LME4 authors, version 1.1.18.1) for the mixed effect linear and logistic regression and the R-package zCompositions (version 1.1.1) [19] for the imputation of left censored data.

## 3. Results

### 3.1. Study Population

A total of 5667 blood samples were measured for both cTnT and cTnI in the period from 27 September 2013 to 11 February 2014 (Table 1). A total of 2318 blood samples came from female patients, while 3349 were from male patients. In addition, 42.5% of the patient cases were female and 57.5% male, which means for the further statistical analysis that the male samples predominate. The median age is 71 for women and 67 for men, and the age distribution proved homogeneous. The troponin values for cTnI and cTnT were distributed over almost the entire linearity range of the assays. On average, men showed higher cTnI (16 ng/L) and cTnT values (22 ng/L) than women (cTnI: 10 ng/L; cTnT: 14 ng/L). The eGFR of men (median 73 mL/min/1.73 m^2^) was similar to that of women (71 mL/min/1.73 m^2^), which, in formal, means for most patients an impaired kidney function. The descriptive data show a relatively homogeneous distribution between men and women, which underpins the significance of the following gender-specific analyzes.

### 3.2. Influencing Factors of cTnT and cTnI

Using univariate and multiple linear mixed effect models, the baseline characteristics sex, age, eGFR, and creatinine were tested for potential influence on the cTnI and cTnT levels (Table 1). In the univariate models, age, creatinine, eGFR and gender have significant influence on cTnT and cTnI concentrations. In the multi-variable model, only the parameter “age” as an influencing factor remained.

### 3.3. Pairwise Correlations for cTnT, cTnI and Creatinine

**Figure 2:**
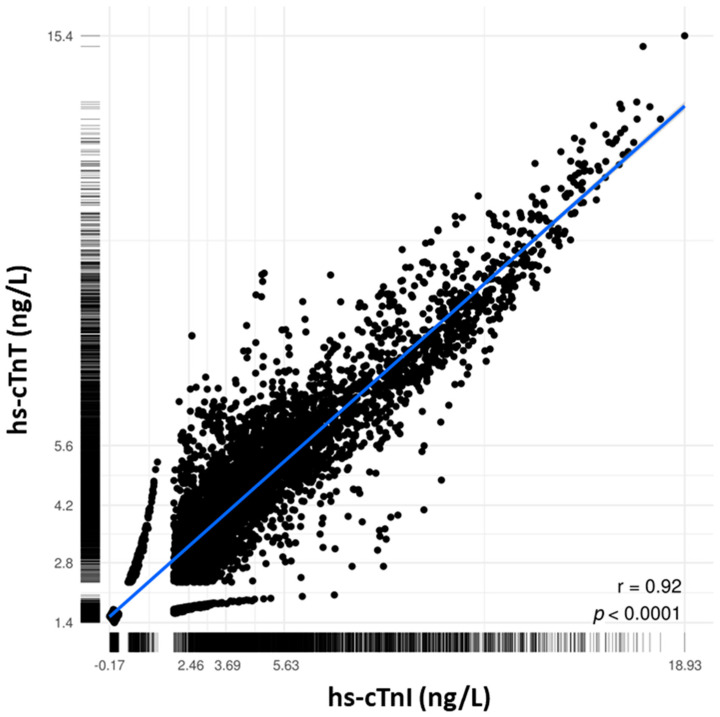
Correlation analysis between cTnT and cTnI. Scatterplot of hs-cTnT and hs-cTnI measurements in all blood samples (*n* = 5667). The limit of detection was set at 5 ng/L for hs-cTnT and 1.9 ng/L for hs-cTnI. The blue line is the regression line. Pearson correlation coefficient is given. Axes are log scaled.

A good positive linear correlation (r = 0.92) was shown between cTnT and cTnI (Figure 2). A weak positive correlation was found between the cTnT concentration (Supplementary Figure S1A) and the creatinine concentration (r = 0.33). This was more pronounced than that between cTnI (Supplementary Figure S1B) and the creatinine concentration (r = 0.24).

### 3.4. Discrepancy between cTnT and cTnI

In the comparison of the measurement results, analyses for cTnI were done with both the gender-specific and the unisex cutoff (Table 2). An overview of the distribution of cTn concentrations between the genders and in patients with an eGFR > 60 and ≤ 60 mL/min/1.73 m^2^ with depiction of the various used cutoffs can be found in the supplements (Supplementary Figure S2). Concentrations above the threshold were considered positive (cTnT+; cTnI+). Comparing the results of cTnT and cTnI in all blood samples (*n* = 5667) and using the cTnI unisex cutoff, it appeared that 83.81% were congruent. However, 13.99% showed a cTnT+ and cTnI− result. The discrepancy for cTnI+ and cTnT− samples was only 2.19%. According to gender (*n* (male) = 3349; *n* (female) = 2318), discrepant results were only slightly higher in men (cTnI−/cTnT+: 14.45%; cTnI+/cTnT−: 2.24%) than in women (cTnI−/cTnT+: 13.33%; cTnI+/cTnT−: 2.11%).

However, when using gender-dependent cutoffs for cTnI, the situation was different: Only 75.03% of the samples showed a congruent result. The discrepant results increased in the group of cTnI−/cTnT+ patients (23.84%) and, interestingly, decreased in the cTnI+/cTnT− group (1.13%) compared to unisex cutoff use. An explanation may be provided by the gender-related evaluation of test results: Here, the discrepant results of the cTnI−/cTnT+ group for men were higher (29.91%) than using the unisex cutoff, whereas it hardly differed in women (15.05%). In contrast, the percentage of the cTnI+/cTnT− group was lower than that of the unisex (male: 0.68%; female: 1.76%). Because the male cutoff of cTnI (34.2 ng/L) is above and the female one (15.6 ng/L) clearly below the unisex cutoff (26.2 ng/L), this could lead theoretically to overdiagnosed men, while the otherwise underdiagnosed women were more frequently detected positive.

CTnT and cTnI concentrations depend on their clearance in the blood and on renal function [20]. In particular, cTnT concentrations show a strong dependence on GFR [21]. In order to rule out the kidney function dependency of the cTn concentrations as cause of the discrepancies described, analyses (Table 2) were carried out again for patient cases of the cohort in which an eGFR > 60 mL/min/1.73 m^2^ was found (*n* = 2473). The relationships concerning the discrepancies were very similar to those in the overall cohort: 20.95% of cTnI−/cTnT+ blood samples using the gender-specific cutoff and 13.18% for the unisex cutoff of cTnI (respectively for cTnI+/cTnT−: 1.33% for gender specific and 2.51% for the unisex cutoff). These very similar relationships show that the dependence of the cTn concentrations on the kidney function is not sufficient to explain the differing results.

### 3.5. Dynamic Analysis of Serial cTn Measurements within 12 h

In order to compare the assessability of cTnI and cTnT in the clinical context of a suspected cardiac diagnosis, comparisons were carried out on dynamic analyses of serial troponin measurements (Table 2). For this purpose, all patients were selected for which at least two troponin measurements were available that were carried out within 12 h (*n* = 1329). These cases were declared positive if at least one cTn concentration was above the cutoff and there was a difference of at least 20% between the two troponin concentrations. Gender-specific and unisex cutoffs were used again for cTnI. This was an attempt to get as close as possible to the acute use of the cTn according to clinical guidelines [2].

Most of the patient cases sorted according to these criteria did not show a positive course of the troponins within 12 h (gender specific cutoff *n* = 948; unisex cutoff *n* = 923). Of all positive courses, approx. 48% were discrepant between cTnT and cTnI (unisex cutoff: *n* = 192; gender specific cutoff: *n* = 185), in which 26% were cTnI+/cTnT− (*n* = 101) and 22% cTnI−/cTnT+ (*n* = 84) when using the gender specific cutoff. When using a unisex cutoff, only 20% were cTnI+/cTnT− (*n* = 83), but 26.8% cTnI−/cTnT+ (*n* = 109). However, the discrepant cases are balanced. The decrease in the cTnI−/cTnT+ cases and the increase in the cTnI+/cTnT− cases when using the gender specific cutoffs can be explained by the fact that the lower cutoff for women achieves more women to be recognized as positive and more men do not count as positive cases due to the increased cutoff for men.

If only the few cases that were really diagnosed as myocardial injury (*n* = 137) are considered in the identified patient cases from the dynamic analysis, 13% of these have discrepant cTn courses (*n* = 18) in the first 12 h. Provided that only the cTnT values were reported to the clinician, the suspected diagnosis of myocardial infarction would have been made earlier in ten cases if cTnI had been reported.

### 3.6. Diagnosis of Study Cohort

Main diagnoses could be determined in 1998 patients (Figure 1). From that, 1816 were treated in hospital, while only 182 were treated in the emergency unit. The majority (*n* = 906) was related to various diagnoses that cannot be categorized uniformly (Table 3). Another large part is due to cardiovascular diseases (*n* = 781), of which 137 showed angina pectoris and 215 an acute myocardial infarction. Furthermore, 54 had unspecific chest pain. The other diagnoses can be summarized under neurological diseases (*n* = 145) and respiratory diseases (*n* = 112).

### 3.7. Specificity and Sensitivity of cTnT and cTnI in Myocardial Infarction Diagnosis

**Figure 3:**
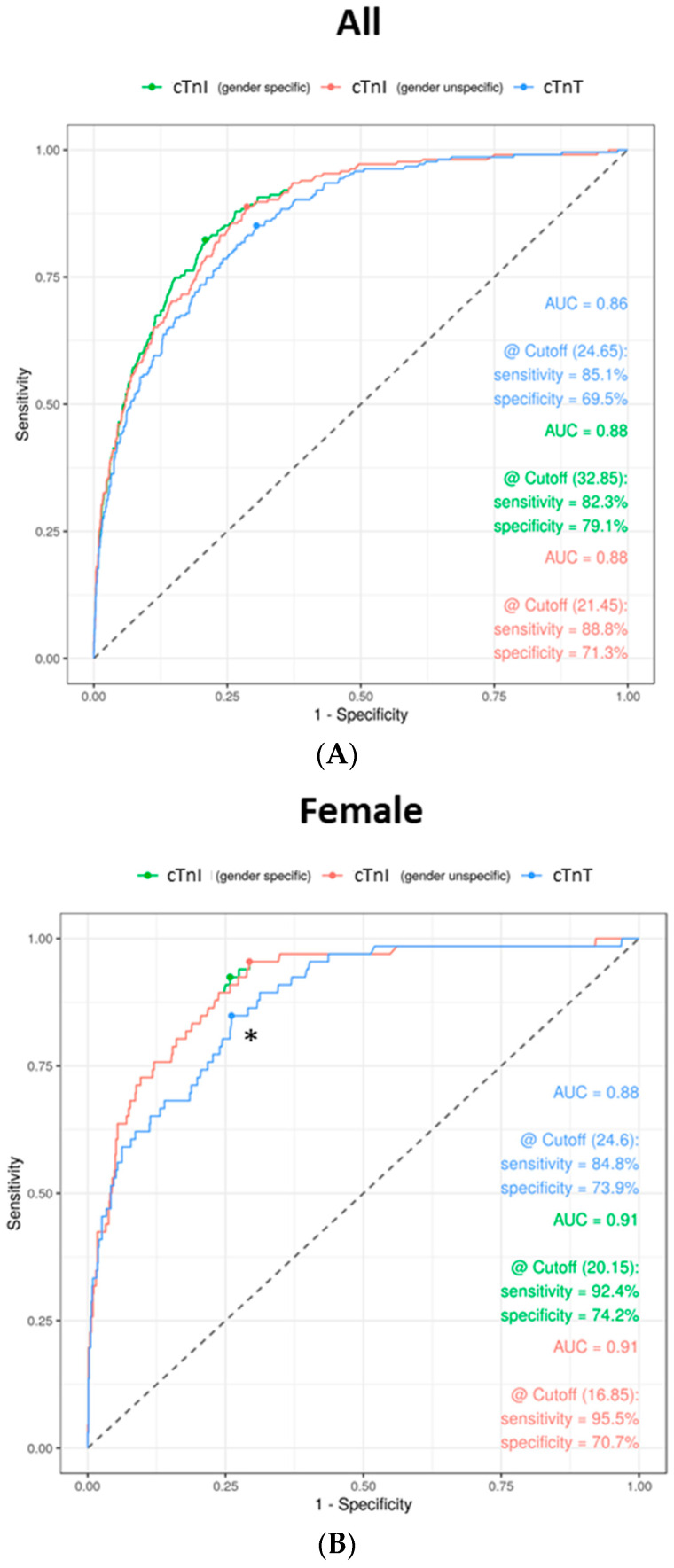

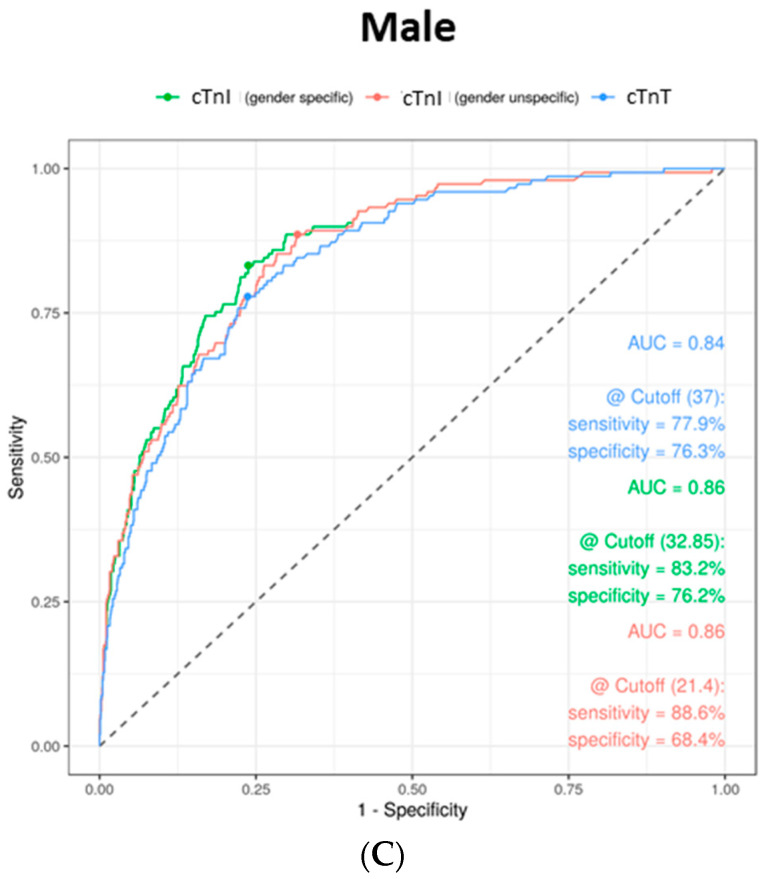
Receiver operating characteristic (ROC) analysis for cTnI and cTnT. ROC curves are shown for all patient cases ((**A**); *n* = 1998) and for female ((**B**); *n* = 806) and male ((**C**); *n* = 1192) distinctly. Analyses are based on the diagnosis of myocardial injury. Green curves are for cTnI with gender-specific cutoffs, red curves for cTnI with gender-unspecific cutoff and blue curves for cTnT. Calculated are area under the curves (AUC), optimized cutoffs (Youden Index) and sensitivities as well as specificities at the calculated cutoffs. * *p* < 0.05 (DeLong’s Test comparing cTnI and cTnT).

In order to investigate the specificity and sensitivity of cTnT and cTnI in the diagnosis of myocardial infarction based on the main diagnoses of the patients in the study cohort, ROC analyses were carried out (Figure 3). For cTnT, highest sensitivity (85.1%) and specificity (69.5%) were determined using a cutoff of 24.65 ng/L. With a unisex cutoff of 21.45 ng/L, cTnI can achieve a sensitivity of 88.8% and a specificity of 71.3% (Figure 3A). The calculated cutoff for cTnT is significantly higher than that recommended by the manufacturer (14 ng/L). In contrast, the threshold for cTnI is close to the value determined by the manufacturer (26.2 ng/L). The AUCs are comparable (cTnT: 0.86; cTnI: 0.88). Furthermore, gender-related calculations were considered separately (Figure 3B,C): In women, cTnT with a cutoff at 24.6 ng/L showed a sensitivity of 84.8% and a specificity of 73.9%. In men, a lower sensitivity (77.9%) with a higher specificity (76.3%) at a concentration of 37 ng/L could be achieved. The AUC is higher in women (cTnT: 0.88; cTnI: 0.91) than in men (cTnT: 0.84; cTnI: 0.86). For cTnI, using a gender-independent cutoff for women of 16.85 ng/L, a sensitivity of 95.5% and a specificity of 70.7% was estimated. In contrast, men with a cutoff of 21.4 ng/L have lower specificity (68.4%) and sensitivity (88.6%) (DeLong’s test: *p* = 0.049). On the other hand, a gender-specific cutoff for cTnI in women (20.15 ng/L) increases the sensitivity to 92.4% and lowers the specificity to 74.2%. In men, a lower sensitivity (83.2%) and sensitivity (76.2%) at a cutoff of 32.85 ng/L could be detected (*p* = 0.089). Overall, it can be seen that cTn shows a higher sensitivity in women and a higher specificity in men when diagnosing myocardial infarction by using gender-dependent cutoffs. The highest results are achieved by cTnT especially with a distinctly higher cutoff than recommended by the manufacturer and especially when considering the gender differences. Overall, cTnI appears to have better specificity than cTnT, with cTnT showing better specificity in men and cTnI better sensitivity in women.

**Figure 4:**
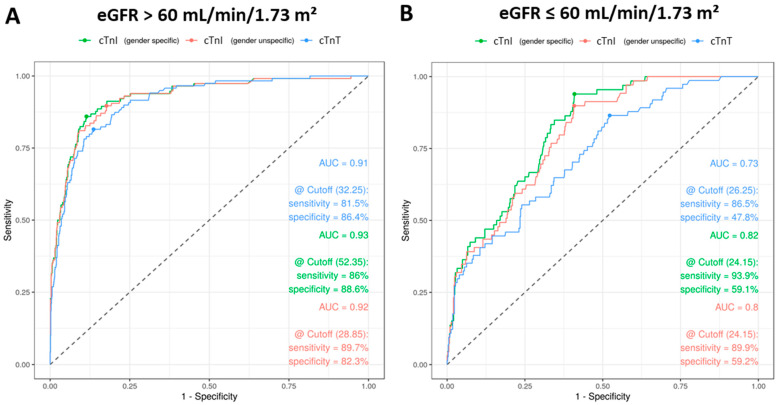
Receiver operating characteristic (ROC) analysis for cTnI and cTnT for patient cases with impaired kidney function. ROC curves are shown for all patient cases with an eGFR > 60 mL/min/1.73 m^2^ (**A**) and ≤ 60 mL/min/1.73 m^2^ (**B**) distinctly. Analyses are based on the diagnosis of myocardial injury. Green curves are for cTnI with gender-specific cutoffs, red curves for cTnI with gender-unspecific cutoff and blue curves for cTnT. Calculated are area under the curves (AUC), optimized cutoffs (Youden Index) and sensitivities as well as specificities at the calculated cutoffs.

Furthermore, the influence of the eGFR on the sensitivity and specificity of the cardiac troponins was examined (Figure 4). When considering all patients with an eGFR > 60 mL/min/1.73 m^2^, cTnT showed a sensitivity of 81.5% and a specificity of 86.4% at a cutoff value of 32.23 ng/L (Figure 4A). cTnI had a sensitivity of 86% (unisex: 89.7%) and a specificity of 88.6% (unisex: 82.3%) using gender-specific cutoffs at a cutoff of 52.35 ng/L (unisex: 28.85%). The AUC was very high for both cTnT (0.91) and cTnI (0.93). When considering only the patients with an eGFR ≤ 60 mL/min/1.73 m^2^ (Figure 4B), clear differences can be seen (DeLong’s test: *p* < 0.001): The specificity falls for cTnI with a gender-specific cutoff value of 24.15 ng/L (unisex: 24.15 ng/L) to 59.1% (unisex: 59.2%), and the sensitivity increases to 93.9% (unisex: 89.9%). With cTnT the sensitivity increases to 86.5%, and the specificity falls to 47.8% with a cutoff of 26.25 ng/L. The AUC falls for both, cTnT (0.73) and cTnI (0.8). In summary, the clear dependency of the cardiac troponins on kidney function can be seen: Reduced kidney function significantly lowers the specificity of the diagnosis with cardiac troponins, while sensitivity is increased. However, it can be seen that the specificity of cTnI is less dependent on kidney function than that of cTnT. Interestingly, it may be noticed that for patients with normal kidney function, the highest sensitivities and specificities result from higher cutoffs than those recommended by the manufacturers.

## 4. Discussion

Discrepancies of cTnT and cTnI results may lead to differences in the assessment of thoracic pain and differential diagnosis of myocardial infarction.In the present study, the diagnostic differences between the high-sensitive assays cTnT (Roche) and cTnI (Abbott) were evaluated. The questions raised in the introduction are discussed as follows.

(I) We firstly analyzed the number of discordant cTnT/cTnI classifications (with respect to each single sample) considering gender-independent cutoffs of both markers. 16.18% of all samples included showed discrepant results. Of these, 13.99% were cTnI−/cTnT+. Compared to all positive samples, 27.41% were discrepant between cTnI and cTnT. What would that clinically mean? In hospitals with labs using the cTnT assay, attending doctors would, e.g., diagnose a suspected AMI while in others, using the cTnI assay, physicians would exclude this diagnosis. In a relevant percentage of patients with differential diagnosis AMI, the assay chosen might decide about the patient’s diagnosis. At the same time, patients without AMI and potentially ‘falsely’ elevated troponin may be overdiagnosed.

In a second step, we repeated the analysis using gender-specific ranges for cTnI in comparison with the gender-independent range for cTnT (gender-specific ranges for TnT were not given by the manufacturer). When using gender-specific cutoffs for cTnI, the rate of discordant cases of cTnI−/cTnT+ results increased to 23.84% of all included samples. Related to all positive samples, 43.07% of the results were discordant (only men: 49.01%, only women: 32.58%). The main reason here is the widening of the discrepancies among men (to 29.91% related to all samples). This is due to the fact that the gender-specific cutoffs of cTnI (men: 34.2 ng/L; women: 15.6 ng/L) are higher for men and lower for women than the gender-independent 99th percentile URL (26.2 ng/L). As a result, otherwise overdiagnosed men are now declared as cTnI− and otherwise underdiagnosed women as TnI+. However, this effect is more pronounced in men than in women. This suggests a clear clinical difference in the assessment of cTn that would result from using gender-specific cutoffs. Studies examined this relationship with regard to the performance and prognostic significance of gender-specific cutoffs for cTnT and cTnI [14]. One effect seems to be particularly evident in women: for cTnI, the use of gender-specific cutoffs led to a significant increase in the diagnosis of myocardial injury in women, while in men, this diagnosis decreased only marginally [22,23,24]. This finding matches with our retrospective data. Nevertheless, this does not seem to show any effect on the identification of risk patients, on prognosis or 1-year outcome [23,25,26]. On the other hand, there are also data that support the predictive value of increased cTnI in gender-dependent cutoffs in women [27]. Few studies have investigated these aspects for cTnT, but also here, it appears that gender-dependent cTnT cutoffs would increase the myocardial injury diagnosis in women and decrease it in men, since the general cutoff (14 ng/L) is set too high for women and too low for men [28,29,30]. McRae et al. assume an improved specificity of cTnT when using gender-dependent cutoffs [31]. Nevertheless, until today, no improvement in outcomes [29] or altered risk prediction [32] is described for using gender-specific TnT cutoffs. In summary, the use of gender-specific cutoffs in cTnI leads to an increase in the diagnosis of myocardial damage in women and a decrease in men, which would probably also apply to cTnT. So far, however, there is no clear evidence whether gender-based cutoffs should be clinically used and still no gender-specific cutoffs are available for cTnT. As shown, gender-specific cutoffs cannot fully adequately explain the discrepant results between cTnT and cTnI.

(II) The initially mentioned discrepancies between cTnT and cTnI in diagnosing myocardial injury were based on one-time measurements. However, this does not correspond to the assessment in everyday clinical practice [2] in which a rise and/or fall of cTn values with at least one value above the 99th percentile URL is set. We wanted to analyze whether the discordant results would be reproduced with serial measurements under consideration of troponin kinetics. No specific declaration of the extent of a significant rise or fall of cTn values is given in the existing guidelines [2]. Therefore, we defined a cTn increase or decrease of at least 20% compared to the initial concentration as significant ‘kinetics’ [33]. In this study, only a limited number of patient cases met the criteria mentioned above, but it could be shown that the discrepant cases were 47% of all positive cases.

Compared to all cases met our criteria, only approx. 14% were discrepant between cTnI and cTnT, regarding to the fact that approx. 70% were negative according to our criteria. Using gender-specific cutoffs, only a little shift is shown from cTnI−/cTnT+ patient cases to more cTnI+/cTnT− patient cases. Interestingly, using gender-specific or unisex cutoffs, the proportion of discrepant findings was balanced between cTnI−/cTnT+ and cTnI+/cTnT− cases. This could suggest that the use of the kinetic cTn course would quasi nihilate the difference between cTnI and cTnT results and discrepant findings would be equally often on both sides. However, from this subgroup, only 137 patients were really diagnosed as myocardial infarction, and only 18 patients were in the group with discrepant findings. This suggests that many diagnoses were probably only made through further troponin measurements or through clinical symptoms that cannot be shown here. Moreover, many positive courses were not diagnosed as myocardial infarction, which may be due to the fact that there were possible differential diagnoses of myocardial infarction in the population under consideration, which could also lead to increased cTn.

(III) Finally, we used ROC analyses to compare the diagnostic performance of cTnT and cTnI and to define gender-specific cutoffs. The AUC for cTnT was only slightly below that of cTnI, but the specificity of cTnT was significantly lower than that of cTnI. It is remarkable that when looking at the genders, the sensitivity of cTnI increases in women and the specificity of cTnT in men diagnosing myocardial infarction. This correlates with the above data regarding discordant results between cTnI and cTnT: using gender-dependent cutoffs, the rate of women diagnosed with myocardial injury would increase and that of men would decrease. The highest sensitivity and specificity for cTnI in women are reached by a calculated cutoff of 16.95 ng/L, for men of 21.4 ng/L, for cTnT for women of 24.6 ng/L and for men of 37 ng/L. Studies that define gender-specific cutoffs for cTnT are few [14]. Here, sex-specific 99th percentile URLs were defined in healthy populations: Franzini et al. suggested a cutoff of 23.2 ng/L for men and 10.2 ng/L for women [34]. Saenger et al. found a cutoff of 15.5 ng/L for men and 9 ng/L for women [35]. Overall, there is no consensus, as some studies found 99th percentile URLs above the community cutoff (14 ng/L), some below. When choosing a lower female cutoff below 14 ng/L, it should be considered that the LOD of the Roche assay is 5 ng/L, and there is a problem with fulfilling the criteria for a high-sensitive assay, according to that the assay should measure a cTn concentration in at least 50% of a healthy population with a result above the LOD and below the 99th percentile URL [7]. Again, the slightly higher specificity of cTnI compared to cTnT is known [36].

The influence of kidney performance on troponin levels was analyzed. A positive correlation with creatinine concentration was found for both cTnT and cTnI, whereby the creatinine effect was more pronounced for cTnT, as already observed by Monneret et al. [21]. The influence of kidney performance on the 99th percentile URL of cTnT and cTnI as well as its assay-dependence are well known [20]. This knowledge underlines the importance and superiority of Tn kinetics in comparison to the absolute concentration of a single sample for the diagnosis of myocardial infarction without ST-segment elevation [37]. Using the manufacturer’s threshold, specificity of cTnT decreases with increasing renal insufficiency [38]. With the help of ROC analyses, we could show in this study that—while maintaining sensitivity—specificity of cTnT and also cTnI decrease significantly in patients with an eGFR < 60 mL/min/1.73 m^2^. The meaningfulness of cTnT is expressively more limited (AUC 0.73) than that of cTnI (AUC 0.8). These findings could support the use of eGFR-adjusted cutoffs [21] or, more convincingly, the use of diagnostic algorithms with at least two serial measurements, which may correct the diagnostic deficiencies in chronic kidney disease [39].

Some limitations of this study should be noted: Due to the retrospective nature, no follow-up was ascertained for the patients, so that only ‘snapshots’ of clinical progress were included in the analysis. Furthermore, we had no information about comorbidities and the clinical procedures of the patients. In particular, the ROC analyses are only based on the coded diagnoses from the accounting of the hospital, whereby it is known that often not all diagnoses are registered consequently and the suspected diagnoses are missing. However, final diagnoses are also influenced by the cTnT result that was reported. A better approach would be to calculate diagnostic performance that the patients must be adjudicated by independent cardiologist using prespecified diagnostic definitions independently from cTn value. Nevertheless, only 10.8% of our patients had at all an acute myocardial infarction diagnosed. This reduces the value of our ROC analyses in a way. The patient’s renal function is also unknown; only one creatinine concentration per patient case has been included in the analysis, although a temporal biological variability of cTnT and cTnI is known for chronic renal failure [40]. It should be emphasized that the cTnT and cTnI measurements were made directly from the fresh blood sample in succession, and there was no storage in between, so pre-analytical problems were minimized [41].

The discrepant results between cTnT and cTnI should be assessed with regard to the already known biological differences [6,42]. It should also be considered that, unlike cTnI, the cTnT concentration in blood is subject to a physiological diurnal rhythm [43,44]. However, it is described that cTnT can also be expressed in the skeletal muscles in myopathies and that increased concentrations of cTnT could be measured [45,46]. These are all other factors that can be the reason for inconsistent results between cTnI and cTnT.

In summary, we focused in this retrospective study on the different diagnostic statements. It can be said that gender-specific cutoffs for cTnI may increase the sensitivity for women and specificity for men. All these findings confirm that diagnosis of myocardial infarction still based on clinical judgment and results of cTn measurements should be critically examined with regard to the clinical appearance.

## Figures and Tables

**Table 1:** Descriptive data of study cohort (*n* = 5667). Shown are absolute counts with percentage or median with minimum and maximum value. eGFR calculation and eGFR categories are determined according to CKD-EPI requirements. Analyses of influence parameters of cTnI or cTnT: * *p* < 0.05 in univariate linear mixed effect model. ^‡^ *p* < 0.05 in multiple linear mixed effect model analysis.

Parameters		Female	Male *
Blood Samples (counts)		2318 (40.9%)	3349 (59.1%)
Patient cases (counts)		1273 (42.5%)	1719 (57.5%)
Age *^,‡^		71 (18; 98)	67 (18; 97)
cTnI (ng/L)		10 (0.89; 191,822)	16 (0.9; 500,000)
cTnT (ng/L)		14 (2.6; 36,643)	22 (2.6; 43,766)
Creatinine (mg/dL) *		0.81 (0.2; 12)	1.00 (0.27; 14)
eGFR (mL/min/1.73 m^2^) *		71	73
eGFR categories (counts)	G1	338 (24.6%)	487 (25.4%)
	G2	477 (34.8%)	686 (35.7%)
	G3a	226 (16.5%)	324 (16.9%)
	G3b	168 (12.2%)	255 (13.3%)
	G4	131 (9.5%)	122 (6.4%)
	G5	32 (2.3%)	46 (2.4%)

**Table 2:** Contingency table for cTn decisions. cTnT was declared as positive (cTnT+) for all blood concentrations above 14 ng/L. cTnI was declared as positive (cTnI+) for all blood concentrations above 26.2 ng/L for unisex cutoff and for women above 15.6 ng/L, for men above 34.2 ng/L by using gender specific cutoffs. Study population was compared for all blood samples (*n* = 5667), for all patient cases with an eGFR > 60 mL/min/1.73 m^2^ (*n* = 2473) and for all patient cases satisfy the criteria for dynamic analysis (*n* = 1329): At least two serial cTn measurements within 12 h. A patient case was defined as positive in dynamic analysis when between the two serial cTn measurements at least a difference of 20% consisted and at least one measurement concentration was above the defined cutoff. The identified myocardial injury positive cases are related to the cases with cTnI gender specific cutoffs and are settled by the available main diagnosis of the patient cases.

		cTnI− cTnT−	cTnI+ cTnT−	cTnI− cTnT+	cTnI+ cTnT+	*n*
All Blood Samples						
cTnI gender specific cutoffs	All	42.03%	1.13%	23.84%	33.0%	5667
	Male	37.65%	0.68%	29.91%	31.74%	3349
	Female	48.36%	1.76%	15.05%	34.81%	2318
cTnI unisex cutoff	All	40.97%	2.19%	13.99%	42.84%	5667
	Male	36.10%	2.24%	14.45%	47.21%	3349
	Female	48.01%	2.11%	13.33%	36.54%	2318
eGFR > 60 mL/min/1.73 m^2^						
TnI gender specific cutoffs		52.28%	1.33%	20.95%	25.43%	2473
TnI unisex cutoff		51.11%	2.51%	13.18%	33.20%	2473
Dynamic analysis within 12 h						
cTnI gender specific cutoffs		948 (71.33%)	101 (7.59%)	84 (6.32%)	196 (14.74%)	1329
cTnI unisex cutoff		923 (69.45%)	83 (6.24%)	109 (8.20%)	214 (16.10%)	1329
Identified as myocardial injury positive case		58 (6.11%)	10 (9.90%)	8 (9.52%)	61 (31.12%)	137

**Table 3:** Main diagnoses of study population. From 1998 patients (1816 hospitalized, 182 from emergency unit), corresponding main diagnoses were available. The diagnoses are grouped by diseases categories. The absolute counts are given and also the ICD-10 codes.

		ICD-10-Code	*n*
Patient cases			1998
	Emergency unit		182 (9.1%)
	Hospitalized		1816 (90.9%)
Diagnosis			
Cardiovascular diseases			781 (39.0%)
	Essential (primary) hypertension	I10	68 (3.4%)
	Benign hypertension with hypertensive crisis	I10.01	19 (1.0%)
	Malignant hypertension with hypertensive crisis	I10.91	37 (1.9%)
	Angina pectoris	I20	137 (6.9%)
	Acute myocardial infarction	I21	215 (10.8%)
	Acute transmural myocardial infarction of anterior wall	I21.0	22 (1.1%)
	Acute transmural myocardial infarction of inferior wall	I21.1	27 (1.4%)
	Acute transmural myocardial infarction of other sites	I21.2	5 (0.3%)
	Acute transmural myocardial infarction of unspecified site	I21.3	1 (0.1%)
	Acute subendocardial myocardial infarction	I21.4	158 (7.9%)
	Acute myocardial infarction, unspecified	I21.9	2 (0.9%)
	Chronic ischemic heart disease	I25	84 (4.2%)
	Nonrheumatic mitral valve disorders	I34	21 (1.1%)
	Nonrheumatic aortic valve disorders	I35	67 (3.4%)
	Paroxysmal tachycardia	I47	20 (1.0%)
	Atrial fibrillation and flutter	I48	57 (2.9%)
	Heart failure	I50	67 (3.4%)
	Abnormalities of heartbeat	R00	25 (1.3%)
	Atherosclerosis	I70	20 (1.0%)
Neurological diseases			145 (7.0%)
	Intracerebral hemorrhage	I61	20 (1.0%)
	Cerebral infarction	I63	43 (2.2%)
	Intracranial injury	S06	42 (2.1%)
	Syncope and collapse	R55	40 (2.0%)
Respiratory disease			112 (5.6%)
	Malignant neoplasm of bronchus and lung	C34	24 (1.2%)
	Pulmonary embolism	I26	23 (1.2%)
	Pneumonia, organism unspecified	J18	41 (2.1%)
	Other chronic obstructive pulmonary disease	J44	24 (1.2%)
Others			
	Pain in throat and chest	R07	54 (2.7%)
	Other		906 (45.3%)

## Data Availability

The data presented in this study are available on request from the corresponding author. The data are not publicly available due to privacy and ethical reasons.

## Supplementary Materials

The following are available online at https://www.mdpi.com/article/10.3390/jcm10143148/s1. Figure S1: Correlation analysis between cTnT, cTnI and creatinine. Scatterplot of hs-cTnI and creatinine (A) and hs-cTnI and creatinine (B) measurements in all blood samples (*n* = 5667). The blue line is the regression line. Pearson correlation coefficient is given. Axes are log scaled. Figure S2: Distribution of troponin values between the two genders and for patients with an eGFR ≤ 60 and > 60 mL/min/1.73 m². (A) Scatterplot of hs-cTnI and hs-cTnT concentrations for female (orange) and male (red) patients. (B) Scatterplot of hs-cTnI (green) and hs-cTnT (blue) concentrations for patients with an eGFR > 60 and ≤ 60 mL/min/1.73 m² in all blood samples (*n* = 5667). The blue lines demonstrate the different used manufactures cutoffs.
