# Supplementary material for: Inconsistent Findings of Cardiac Troponin T and I in Clinical Routine Diagnostics: Factors of Influence
Source: J Clin Med. 2021 Jul 16;10(14):3148. doi: 10.3390/jcm10143148 (PMC8305654; doi:10.3390/jcm10143148)
Supplement: Supplementary file 1 [file jcm-10-03148-s001.zip › jcm-1280712-supplementary.pdf]

Supplementary Material

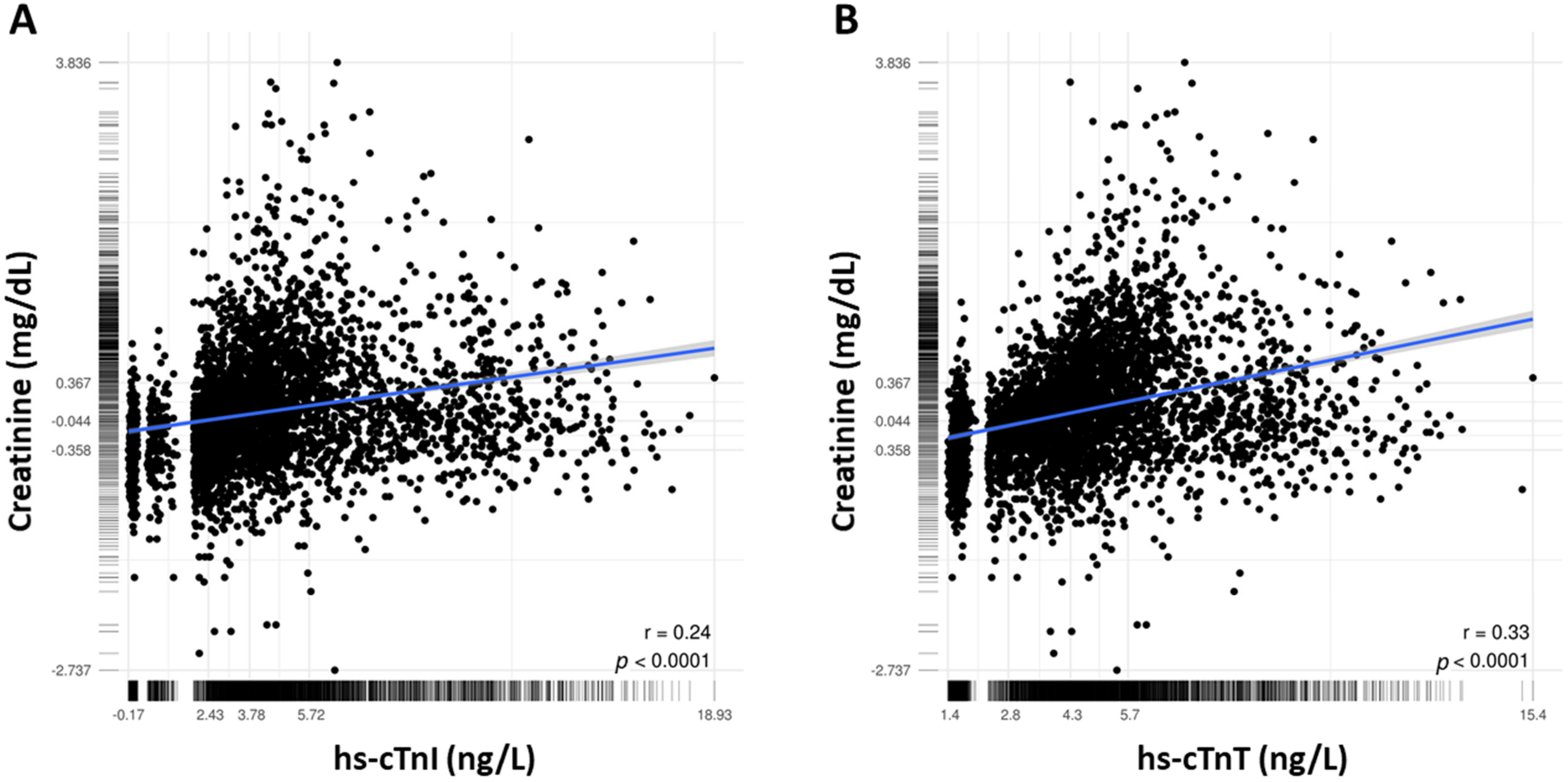

Figure S1. Correlation analysis between cTnT, cTnI and creatinine. Scatterplot of hs-cTnI and creatinine (A) and hs-cTnI and creatinine (B) measurements in all blood samples (n = 5667). The blue line is the regression line. Pearson correlation coefficient is given. Axes are log scaled.

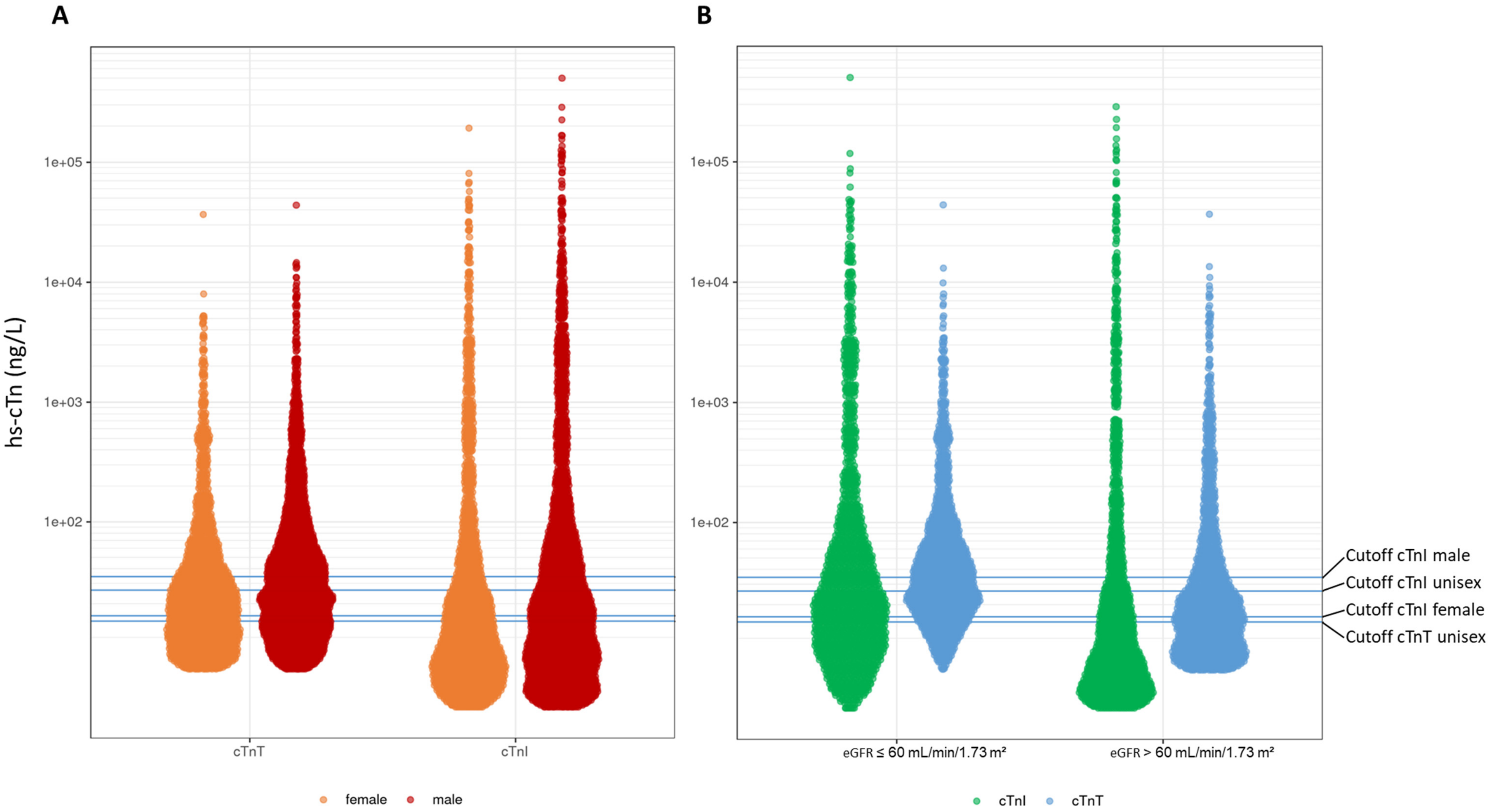

Figure S2. Distribution of troponin values between the two genders and for patients with an eGFR ≤ 60 and >60 mL/min/1.73 m². (A) Scatterplot of hs-cTnI and hs-cTnT concentrations for female (orange) and male (red) patients. (B) Scatterplot of hs-cTnI (green) and hs-cTnT (blue) concentrations for patients with an eGFR >60 and ≤ 60 mL/min/1.73 m² in all blood samples (*n* = 5667). The blue lines demonstrate the different used manufactures cutoffs.
